# Targeting *BRAF* in cancers – from molecular diagnostics to personalized therapy

**DOI:** 10.5114/bta/213740

**Published:** 2025-12-08

**Authors:** Zuzanna Pyc, Rafal Rygiel, Dagmara Michalowska, Marcin Ekiert, Izabela Laczmanska

**Affiliations:** 1 Laboratory of Genomics and Bioinformatics, Ludwik Hirszfeld Institute of Immunology and Experimental Therapy, Polish Academy of Sciences, Wroclaw, Poland; 2 Lower Silesian Center of Oncology, Pulmonology and Hematology, Wroclaw, Poland; 3 Department of Oncology, Faculty of Medicine, Wroclaw Medical University, Wroclaw, Poland; 4 Department of Clinical and Experimental Pathology, Faculty of Medicine, Wroclaw Medical University, Wroclaw, Poland

**Keywords:** *BRAF*, cancer, personalized medicine, tailored therapy

## Abstract

Molecular profiling has become a cornerstone of cancer diagnosis and treatment, with *BRAF* alterations serving as significant markers across various tumor types. The gene encodes a serine/threonine kinase involved in the MAPK/ERK signaling pathway, which regulates cell proliferation and survival. Mutations in *BRAF*, notably the V600 codon substitutions, are among the most common genetic drivers in melanoma and other cancers, including thyroid, colorectal, and non-small cell lung cancer. *BRAF* mutations are categorized into three functional classes (class I–III), each with distinct activation mechanisms and therapeutic implications. Current targeted therapies – primarily BRAF and MEK inhibitors, including the first FDA-approved anti-*BRAF* tumor-agnostic therapy – are most effective in cancers harboring the class I V600E mutation. However, the emergence of resistance to BRAF inhibitors has driven the development of next-generation inhibitors and combination treatments. Furthermore, innovative immunotherapy-based treatments have demonstrated synergistic potential in specific *BRAF*-mutated malignancies. Accurate molecular diagnostics are crucial in cancer treatment; therefore, numerous molecular diagnostic methods are employed, including next-generation sequencing (NGS), quantitative PCR, droplet digital PCR, Sanger sequencing, and fluorescence *in situ* hybridization (FISH). NGS, particularly comprehensive genomic profiling, provides the broadest and most detailed genetic data, although simpler laboratory techniques remain popular due to their accessibility and straightforward protocols. Further research into resistance mechanisms and combination therapies, as well as the integration of circulating tumor DNA (ctDNA) in diagnostics, is needed to fully realize the potential of personalized treatment in *BRAF*-driven tumors.

## Introduction

Molecular profiling of tumor cells has become a standard component of cancer diagnostics. Depending on the cancer type, genetic test results are significant for histological diagnosis, prognosis, and, in an increasing number of malignancies, prediction of targeted treatment – including tumor-agnostic therapies. Advances in technology for identifying genetic changes at the DNA, RNA, and epigenetic levels, along with an improved understanding of cancer cell biology, have enabled the implementation of new molecular tests and effective targeted therapies in oncology (Rulten et al. 2023; Gouda et al. 2023).

The mitogen-activated protein kinase (MAPK) pathway was among the first to be identified as containing protein components that could serve as targets for therapy. From the discovery of RAS proteins in the 1960s to the elucidation of the RAS/RAF/MEK (MAPK/ERK) pathway in the 1980s and the introduction of RAS/RAF/MEK inhibitors for cancer treatment in the early twenty-first century, extensive research has been conducted. The results of these studies are now considered foundational and are routinely applied in clinical oncology (Bahar et al. 2023; Gouda et al. 2023).

Aside from *KRAS*, the *BRAF* gene is the most frequently mutated component of the MAPK/ERK pathway. This signaling cascade plays a crucial role in regulating cell growth, division, and survival (Yi et al. 2022). Alterations in the *BRAF* gene that affect protein functionality and are clinically actionable can significantly influence cellular behavior, potentially leading to malignant transformation. *BRAF* pathogenic variants are found in over 50% of melanomas and up to 9% of all human cancers – most commonly thyroid, prostate, gastric, breast, colorectal, and lung cancers. Consequently, *BRAF* has become a biological target for anticancer monotherapy, combination therapy, and a promising candidate for tumor-agnostic treatment (Poulikakos et al. 2022; Gouda et al. 2023; Dillon et al. 2021; Owsley et al. 2021; Roa et al. 2024).

## *BRAF* function in carcinogenesis and tailored therapy

The *BRAF* gene, located on chromosome 7q34, encodes the BRAF proto-oncogene protein kinase. Along with ARAF and CRAF, BRAF belongs to the family of serine/threonine protein kinases known as RAF, which are integral to the MAPK/ERK signaling pathway (Yi et al. 2022). RAF kinases exist in the cytoplasm as inactive monomers due to autoinhibition mediated by intramolecular contacts between their regulatory and catalytic domains, as well as binding to the 14-3-3 dimer. In response to an activation signal, RAF proteins interact directly with GTP-bound RAS at the plasma membrane. This interaction disrupts RAF autoinhibition and promotes dimerization, leading to kinase activation (Martinez Fiesco et al. 2022).

The most common *BRAF* mutations are substitutions of the valine residue (Val, V) at codon 600 (Val600, V600), which stimulate RAS-independent monomeric activation of BRAF kinase. Codon V600 accounts for approximately 90% of all *BRAF* mutations (Pelosi et al. 2024). Mutations involving this codon are classified as class I mutations. Among the various substitutions at this site, the most frequent – p.Val600Glu (V600E) is a major driver in the development of melanoma, lung cancer, thyroid cancer, and colorectal cancer. Other variants include p.Val600Lys (V600K), p.Val600Leu (V600L), p.Val600Arg (V600R), and p.Val600Asp (V600D), in which valine at position 600 is replaced by lysine, leucine, arginine, or aspartic acid, respectively. These variants are also significant risk factors for melanoma, thyroid carcinoma, multiple myeloma, colorectal adenocarcinoma, and cancers of unknown primary (Owsley et al. 2021).

BRAF serine/threonine kinase inhibitors – such as vemurafenib, dabrafenib, and encorafenib – compete with ATP for binding to the intracellular domain of active BRAF^V600^ monomers. These agents stabilize the αC-helix in the inactive OUT conformation and are therefore referred to as αC-OUT inhibitors (Cotto-Rios et al. 2020). However, in cells harboring RAS mutations, these inhibitors promote wild-type RAF (RAF^wt^) dimerization, thereby enhancing ERK pathway activation – an effect known as the “RAF inhibitor paradox”. This paradox can be mitigated through the use of MEK inhibitors (Roa et al. 2024; Pelosi et al. 2024; Martinez Fiesco et al. 2022; Durrant et al. 2017).

Several mechanisms of cancer cell resistance to RAF inhibitors have been identified, including RAS mutations, *BRAF* amplification, expression of *BRAF*^V600E^ splicing variants, and feedback stimulation of receptor tyrosine kinases and RAS. This phenomenon – reactivation of ERK signaling despite therapy – is frequently caused by RAF protein dimerization. The aforementioned RAF αC-OUT inhibitors, unfortunately, inhibit BRAF dimers poorly, as they can bind only to one subunit of the RAF dimer (Cotto-Rios et al. 2020).

Other *BRAF* mutations, specifically non-V600 variants that also induce functional changes in the protein, are classified as class II or class III. Class II mutations lead to RAS-independent activation of BRAF dimers, while class III mutations suppress BRAF kinase activity while maintaining the ability to bind CRAF via a RAS-dependent mechanism (Owsley et al. 2021).

Class II *BRAF* mutations result in constitutive, RAS-independent activation of BRAF homodimers (BRAF^mut^/BRAF^mut^). This loss of autoinhibitory control promotes oncogenic transformation. Notable examples of class II mutations include p.Lys601Glu (K601E), p.Leu597Gln (L597Q), and p.Gly469Ala (G469A), as well as substitutions at codons G464, G469, K601, and L597 (Śmiech et al. 2020; Durrant et al. 2017). These mutations represent the second most common cause of melanoma among *BRAF* mutation classes, accounting for approximately 12% of *BRAF*-mutated melanomas (Pelosi et al. 2024).

Class II mutations primarily affect the activation segment and P-loop of the BRAF protein and can be further divided into two subcategories: class IIa and class IIb (Śmiech et al. 2020; Pelosi et al. 2024). Class IIa mutations, such as those at codons Leu597 (L597) and Lys601 (K601), disrupt the structure of the protein’s activation loop, whereas class IIb mutations, including those at codons Gly469 (G469) and Gly476 (G476), alter the glycine-rich region of the kinase domain (Pelosi et al. 2024).

TAK-632, LY3009120, and AZ-628 are examples of next-generation RAF inhibitors that stabilize the αC-helix in the active IN conformation (αC-IN inhibitors). These inhibitors bind not only to monomers but also to each subunit within RAS-dependent RAF dimers, thereby promoting catalytic inhibition of both. However, by enhancing RAF binding to active RAS, αC-IN inhibitors can also promote RAF dimerization and subsequent ERK pathway activation, which is unfavorable for therapeutic outcomes.

The recently developed RAF αC-OUT inhibitor PLX8394, which selectively degrades BRAF dimers in RAS-independent signaling triggered by *BRAF* mutations, may address this issue when used in combination with αC-IN inhibitors (Cotto-Rios et al. 2020).

Additionally, ponatinib – an FDA-approved tyrosine kinase inhibitor used to treat chronic myeloid leukemia and Philadelphia chromosome-positive (Ph+) acute lymphoblastic leukemia – has been shown to exhibit high affinity for active BRAF monomers and dimers. In cases involving class II *BRAF* mutations, ponatinib can promote the formation of inactive BRAF^mut^/CRAF dimers and BRAF/MEK1/2 complexes. Because this molecule can also induce RAF dimer formation in RAS^mut^ BRAF^wt^ cells, it holds potential for use in melanoma and other *BRAF*-dependent cancers, particularly in combination with MEK inhibitors or immunotherapy (Cotto-Rios et al. 2020; Durrant et al. 2017; Martinez Fiesco et al. 2022).

Class III *BRAF* mutations result in a significant reduction in kinase activity. They are primarily caused by deletions in DNA fragments encoding the DFG motif (residues 594–596; Asp-Phe-Gly), the P-loop, or the catalytic loop. The DFG motif is a highly conserved sequence within the ATP-binding site that coordinates magnesium ion binding. Common class III mutations include p.Gly466 (G466), p.Asn581 (N581), p.Gly596 (G596), and p.Gly594 (G594), which – along with class I substitutions – have been implicated in the development of myeloma, melanoma, and colorectal cancer (Śmiech et al. 2020). BRAF proteins harboring class III mutations cannot activate MEK independently but retain CRAF-binding ability in a RAS-dependent manner; thus, through CRAF activation, they can still promote carcinogenesis (Cotto-Rios et al. 2020; Owsley et al. 2021).

Other known *BRAF* abnormalities include gene fusions with various partners resulting from chromosomal translocations. These fusions create hybrid genes – combinations of fragments from normally separate genes – that, when expressed, lead to the production of aberrant proteins (Ross et al. 2016). Currently known types of *BRAF* fusions are associated with increased proliferation and invasiveness of cancer cells in oncologic patients (Turner et al. 2018). These fusions represent an alternative mechanism of BRAF protein activation and are found in numerous tumor types, most notably melanomas, thyroid cancers, gliomas, pancreatic carcinomas, colorectal cancers, and non-small-cell lung cancers (Ross et al. 2016).

*BRAF* fusions, which frequently occur in melanomas, are a primary driver of oncogenic transformation in cells lacking other known protooncogenic mutations. Such cases are classified as pan-negative melanomas (Turner et al. 2018).

*BRAF* gene fusions typically involve the C-terminus of *BRAF* (exons 9–18, encoding the kinase domain) and result in the loss of the regulatory N-terminal domain. This alteration leads to autophosphorylation, constitutive dimerization, and persistent activation of BRAF kinase, thereby continuously stimulating the MAPK pathway (Yi et al. 2022; Turner et al. 2018). The most common fusion partners of *BRAF* include *AKAP9* (prevalent in melanomas), *NUP214* (prevalent in lung carcinomas), and *KIAA1549* (prevalent in gliomas, sarcomas, and breast cancers) (Ross et al. 2016).

Another type of aberrant alteration affecting *BRAF* activity is gene amplification, which occurs when abnormal replication of the *BRAF* gene results in more than two copies per cell. This amplification can increase BRAF protein activity, posing a particular risk in the case of mutated proto-oncogenes such as *BRAF. BRAF* amplification is frequently responsible for resistance of neoplastic cells to cytostatic therapies. In tumors harboring class I mutations, amplification of BRAF^V600E^ represents a major mechanism of resistance to MEK inhibitors (Sale et al. 2019).

Vemurafenib was the first BRAF inhibitor approved in the United States for the treatment of late-stage melanoma in 2011. Since then, these drugs have become an integral part of advanced melanoma therapy. Expanding knowledge of *BRAF*’s role in various cancers led the U.S. Food and Drug Administration (FDA) to approve a tumor-agnostic therapy in 2022, involving the use of the BRAF inhibitor dabrafenib in combination with the allosteric MEK inhibitor trametinib for solid tumors harboring the *BRAF*^V600E^ mutation, regardless of tumor type (Weiss et al. 2025).

Testing for *BRAF*^V600^ mutations is mandatory in stage III–IV cutaneous melanoma (Amaral et al. 2025) and metastatic colorectal cancer (Cervantes et al. 2024). Anaplastic thyroid cancer, one of the most aggressive solid tumors in humans, has also demonstrated sensitivity to this therapeutic approach. In a phase II, open-label basket trial, 16 patients with *BRAF*^V600E^-positive cancers were treated with the BRAF inhibitor dabrafenib in combination with the MEK inhibitor trametinib. Eleven of these patients showed a favorable clinical response (Subbiah et al. 2018).

## Molecular methods for detecting *BRAF* alterations in cancers

*BRAF* gene alterations that affect the BRAF protein include single nucleotide variants (SNVs) – primarily missense variants such as the common V600 mutation – small deletions and insertions (indels), fusions with various genes, and gene amplifications. The prevalence of these clinically significant variants varies by cancer type (Yi et al. 2022; Śmiech et al. 2020). Depending on the nature of the genetic alteration, the optimal diagnostic method may differ.

To detect genetic alterations in cancer cells, DNA or RNA (typically 50–150 ng) is extracted from tumor samples collected during surgery or biopsy, including liquid biopsy. Usually, tissue or cell samples are processed into Formalin-Fixed Paraffin Embedded (FFPE) blocks for subsequent analysis. Pre-analytical factors – such as formalin concentration and pH, fixation temperature, sample thickness, fixation duration, and storage procedures – are critical for obtaining reliable and informative genetic data (Steiert et al. 2023).

The most widely used molecular tool for detecting genetic changes in cancer cells is next-generation sequencing (NGS). NGS enables the simultaneous identification of SNVs, indels, and rearrangements at both the DNA and RNA levels. In contrast, other molecular techniques – such as quantitative PCR (qPCR or real-time PCR) for detecting SNVs, indels, fusions, and amplifications; Sanger sequencing for identifying SNVs and indels; and fluorescence in situ hybridization (FISH) for detecting fusions and amplifications – typically allow examination of only one or a few alterations per assay and are generally used for smaller patient cohorts (Ghoreyshi et al. 2025).

NGS overcomes many of the technological limitations of traditional sequencing methods. It is more cost-effective and enables the generation of a greater volume of data in a shorter time for multiple samples simultaneously (Ghoreyshi et al. 2025). Numerous commercially accessible predesigned panels are used to detect selected types of pathogenic variants specific to particular cancer types.

**Table 1:** RAF inhibitors. According to Cotto-Rios et al. (2020)

No.	Inhibitor type	Inhibitor name	Target
1	αC-OUT	Vemurafenib, dabrafenib, encorafenib	BRAF^V600E/K^ monomers
2	αC-IN	TAK-632, LY3009120, AZ-628	BRAF^V600E/K^ monomers and RAS-dependent BRAF dimers
3	αC-CENTRE	Ponatinib	BRAFV^600E/K^ monomers and RAS-dependent BRAF dimers
4	αC-OUT	PLX8394	BRAFV^600E/K^ monomers and RAS-independent BRAF dimers (dimer breaking)

To gain a comprehensive understanding of the molecular mechanisms underlying cancer in an individual patient, comprehensive genomic profiling (CGP) can be performed. This approach enables the examination of all types of DNA/RNA alterations – such as SNVs, indels, CNVs, and fusions – and the assessment of genomic signatures including total mutational burden (TMB), homologous recombination deficiency (HRD), microsatellite instability (MSI), and loss of heterozygosity (LOH). Compared with other molecular tests, CGP offers a substantially greater potential to yield clinically actionable results, which is critical for developing effective personalized therapies. Although CGP is currently the most informative genetic test, it remains too costly and technically demanding for routine diagnostic use (Volders et al. 2025).

Despite the advantages of NGS, simpler methods with lower diagnostic potential continue to be employed. When testing for known driver mutations, these methods are often preferred because they are faster, more affordable, require fewer devices, and are more accessible for laboratory staff.

qPCR is a modified form of standard PCR that allows real-time quantification of the amplified product. Several manual and fully automated qPCR IVD assays are available for diagnostic use and can detect as little as 0.5% of the *BRAF*^V600E^ sequence within a background of normal DNA (Spagnolo et al. 2015). Most available qPCR assays target only codon 600, sometimes without distinguishing the specific variant detected. The presence of any substitution at codon 600 is generally sufficient to qualify a patient for treatment with first- or second-generation BRAF inhibitors. However, these assays are ineffective for detecting less common class II or class III *BRAF* mutations, even though patients harboring such variants may still derive partial therapeutic benefit (Spagnolo et al. 2015; Makutani et al. 2022).

Another PCR-based test is droplet digital PCR (ddPCR), an extremely sensitive technique capable of detecting as little as 0.005% of a variant sequence in a sample. In this method, PCR amplification is performed simultaneously in thousands of nanoliter-sized droplets, each containing a single DNA molecule. Fluorescent detection enables the determination of the ratio between droplets carrying the specific mutation and those containing wild-type DNA, thereby yielding a quantitative result. However, ddPCR does not provide detailed sequence information – it identifies only the presence or absence of a specific variant at a defined location (McEvoy et al. 2018; Min et al. 2024).

**Table 2:** Selected molecular techniques for cancer diagnosis

Method	Neoplastic material tested	Genetic alterations	Limitations
qPCR	FFPE tissue, fresh tissue, whole blood, bone marrow (DNA/RNA); serum (ctDNA/ctRNA)	SNVs, indels	Usually LOD > 10% of neoplastic cells, > 1–5% mutant DNA; requires a specific probe for a target SNV
Digital (dPCR)	FFPE tissue, fresh tissue, whole blood, bone marrow (DNA/RNA); serum (ctDNA/ctRNA	SNVs, indels	Even 0.1–0.001 copies of mutant DNA/µl for specific SNV; 0.01–0.1% mutant DNA; requires a specific probe for a target SNV
FISH	FFPE tissue, whole blood, bone marrow	Chromosomal rearrangements (e.g. deletions, duplications, amplifications, fusions)	Rearrangements in regions > 40–100 kbp; requires a specific FISH probe for a target region
NGS	FFPE tissue, fresh tissue, whole blood, bone marrow (DNA/RNA); serum (ctDNA/ctRNA)	SNVs, indels, exon/gene deletions/duplications/rearrangements (e.g., fusion)	Usually LOD > 10% of neoplastic cells, > 1–5% mutant DNA, high-throughput
Sanger sequencing	FFPE tissue, fresh tissue, whole blood, bone marrow (DNA/RNA)	SNVs, indels	Usually LOD > 20% of variant presence

FISH is a molecular cytogenetic technique that uses fluorescently labeled probes complementary to a specific sequence in a genome. *BRAF* fusions can be assessed using dual-color break-apart or fusion probe sets. The fluorescence signal pattern observed in interphase nuclei enables the detection of *BRAF* gene fusions with different partners (one partner per assay) or, when a break-apart *BRAF* probe is used, the identification of *BRAF* involvement in chromosomal rearrangements. For both probe types, it is not possible to determine whether a transcriptionally active fusion product is produced. Although FISH is highly sensitive and capable of detecting even single cells with rearrangements, a specific cutoff value must be established for each probe set to ensure accurate interpretation (Ghosh et al. 2022; Wang et al. 2018).

Sanger sequencing has long been regarded as the gold standard in molecular biology. For small-scale sequencing, such as hotspot screening, fluorescently labeled dideoxynucleotides are used to terminate DNA strand elongation at specific bases. Its high accuracy and simplicity make it a reliable confirmatory method when results from other molecular tests are unclear or inconsistent. Sanger sequencing can detect both SNVs and indels; however, due to the genetic heterogeneity of cancer tissues, the relative signal intensity of variant peaks may be low. The sensitivity threshold of this method typically requires a variant frequency above 15–20%, which limits its suitability for cancer diagnostics (Cheng et al. 2021).

**Figure 1:**
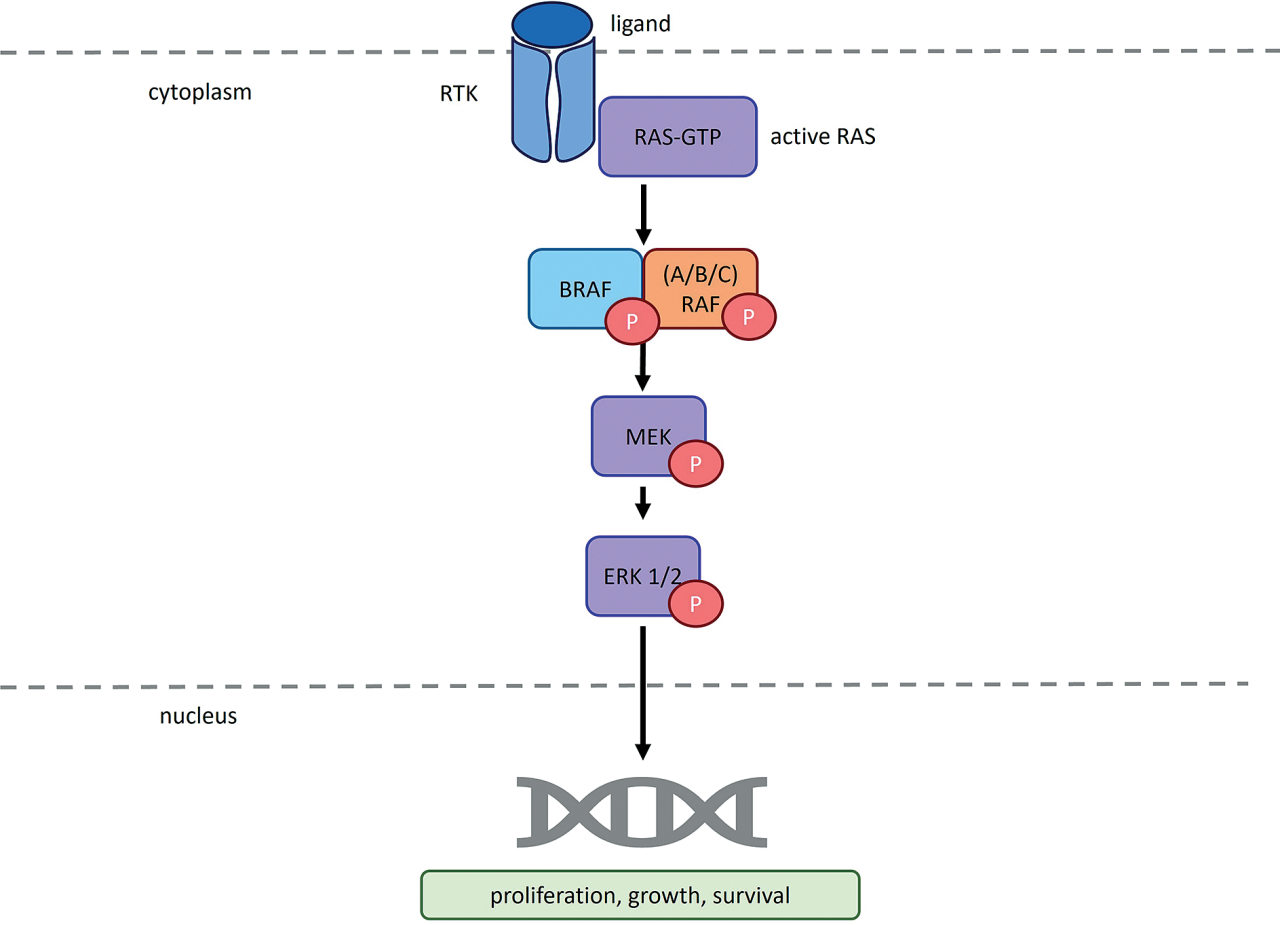
RAS/RAF/MEK/ERK pathway as it functions in cells lacking *BRAF* gene mutations. RTK – receptor tyrosine kinase; RAS – rat sarcoma virus protein; GTP – guanosine-5′-triphosphate; BRAF – serine/threonine-protein kinase B-Raf; ARAF – serine/threonine-protein kinase A-Raf; CRAF – serine/threonine-protein kinase C-Raf; P – phosphorus; MEK – mitogen-activated protein kinase; ERK1/2 – extracellular signal-regulated kinase 1/2. According to Planchard et al. (2024) and Smalley and Smalley (2018)

**Figure 2:**
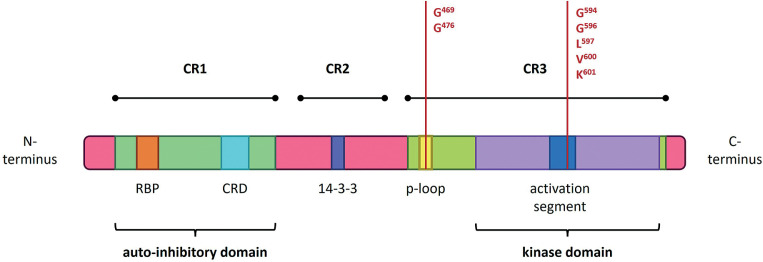
Structure of the *BRAF* gene, showing coding protein domains and the most frequent mutation. CR1/2/3 – conserved region 1/2/3; RBD – RAS-binding domain; CRD – cysteine-rich domain. According to Liu et al. (2020) and Yu et al. (2022)

**Figure 3:**
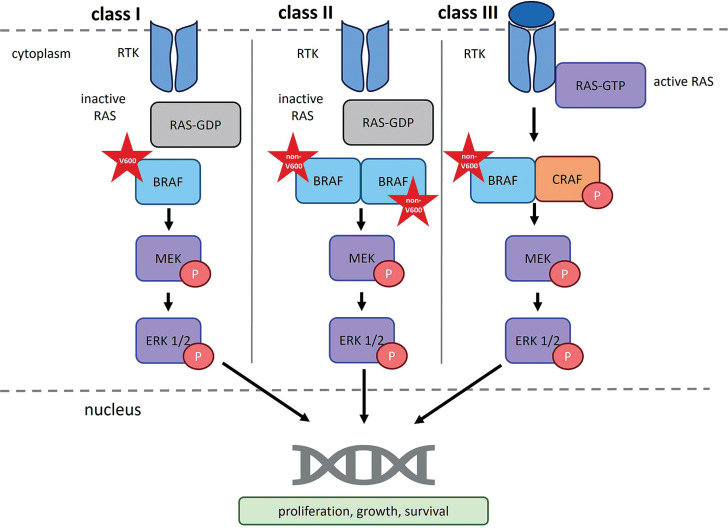
RAF dimerization in normal and oncogenic signaling. RTK – receptor tyrosine kinase; RAS – rat sarcoma virus protein; GDP – guanosine-5′-diphosphate; GTP – guanosine-5′-triphosphate; BRAF – serine/threonine-protein kinase B-Raf; CRAF – serine/threonine-protein kinase C-Raf; P – phosphorus; MEK – mitogen-activated protein kinase; ERK1/2 – extracellular signal-regulated kinase 1/2). According to Bellio et al. (2021), Durrant and Morrison (2017), Martinez Fiesco et al. (2022), and Planchard et al. (2024)

The use of circulating tumor DNA (ctDNA) in cancer diagnostics may, in the future, significantly shorten the time required for molecular testing and serve as a valuable tool for detecting early metastatic stages, particularly when tumors are too small to be visualized through imaging methods. Currently, ctDNA analysis functions primarily as a complementary tool to FFPE tissue examination and represents the only viable option for molecular testing in patients with cancers located in anatomically inaccessible sites (Sobczuk et al., 2022). Published studies have demonstrated a high concordance between *BRAF* testing results obtained from FFPE tissues and ctDNA, with agreement rates ranging from 70 to 90%, underscoring its strong potential for future diagnostic applications (Klein-Scory et al. 2025; Sobczuk et al. 2022).

## Perspectives

Molecular testing of the cancer genome – including the *BRAF* gene – has become a cornerstone of modern oncology, offering tangible prospects for truly personalized treatment strategies. The therapeutic scope of *BRAF* inhibition, through the use of BRAF and MEK inhibitors, has expanded from melanoma-specific applications to a pan-cancer context, positioning *BRAF* as an emerging tumor-agnostic biomarker.

Beyond melanoma and lung cancer, *BRAF*^V600^ mutations are also observed in a range of tumor types, including colorectal, thyroid, biliary tract cancers, and gliomas. Moreover, the spectrum of therapeutic strategies targeting the *BRAF* pathway continues to grow, with numerous agents currently under evaluation across various stages of clinical development. Data from *clinicaltrials.gov* indicate that more than 300 clinical and preclinical studies related to *BRAF* alterations in cancer have been completed, while over 100 trials remain active and continue to recruit patients.

Novel therapeutic approaches are being explored, including inhibitors directed against non-V600 mutations. Ongoing research is also focused on optimizing drug properties – particularly pharmacokinetic profiles and blood–brain barrier penetration – to enhance the efficacy of *BRAF* inhibitors (Gouda et al. 2023).

Molecular profiling of *BRAF* has primarily been associated with the selection of targeted therapies, particularly BRAF and MEK inhibitors, which have significantly improved clinical outcomes in patients with *BRAF*-mutant tumors. However, recent findings suggest that *BRAF* status may also influence the tumor microenvironment and immune response, thereby affecting the efficacy of immunotherapy. Studies indicate that combining immune checkpoint inhibitors with BRAF and MEK inhibitors can enhance antitumor activity, suggesting that *BRAF* may play a key role in modulating immunotherapy response. This combination has shown promise as a therapeutic strategy for *BRAF*-mutant colorectal cancers (Tak et al. 2025). While BRAF and MEK inhibitors demonstrate synergistic potential with immunotherapy in melanoma, this combination remains less effective in lung cancer. The biological characteristics of *BRAF*-mutant tumors are influenced by their tissue of origin, co-occurring genetic alterations, and the surrounding tumor microenvironment. A major obstacle in BRAF and MEK-targeted therapy is the development of resistance, often resulting from additional mutations in the MAPK pathway or the upregulation of alternative signaling pathways like PI3K/AKT (Mechahougui et al. 2025).

Further comprehensive molecular analyses are needed to elucidate the role of *BRAF* mutations in cancer biology and therapeutic response, particularly for optimizing targeted strategies and overcoming resistance mechanisms. The continued development of novel therapeutic approaches – such as ERK1/2 inhibitors and next-generation RAF inhibitors – will be critical to advancing personalized oncology. Equally important is the refinement of diagnostic tools, including *BRAF* profiling through ctDNA, which enables noninvasive monitoring and supports timely therapeutic decision-making.

Together, these advancements will enable the transition from standardized treatment strategies toward truly personalized oncology – tailored to each patient, or even to an individual tumor cell line.
